# Prevalence and Viability of *Toxocara* spp. Eggs in Soil of Public Parks in Northwestern Mexico

**Published:** 2020

**Authors:** Alexis Israel VARGAS NAVA, Nohemí CASTRO DEL CAMPO, Idalia ENRÍQUEZ VERDUGO, Jesús José PORTILLO LOERA, Claudia Leonor BARRAZA TIZOC, Soila Maribel GAXIOLA CAMACHO

**Affiliations:** Faculty of Veterinary Medicine and Zootechnics, University Autonomous of Sinaloa, Culiacan, Sinaloa, Mexico

**Keywords:** *Toxocara* spp., Public parks, Prevalence, Viability

## Abstract

**Background:**

*Toxocara* spp. is a zoonotic parasite that can infect human; children are the largest group at risk of infection. Therefore, this study aimed to determine the prevalence and viability of *Toxocara* spp. eggs in the soil of public parks.

**Methods:**

Overall, 1180 soil samples from 236 public parks in four sectors of the city of Culiacan were collected at random, between Jun and Dec, 2013. The presence of *Toxocara* spp. eggs was determined by light microscopy using a centrifugation-flotation technique and viability by trypan blue staining technique.

**Results:**

Of the 236 parks sampled, 18 were positive to *Toxocara* spp. resulting in a prevalence of 7.6% and viability of 94.4% with a *P*<0.05. Detection of *Toxocara* spp.in soil samples was 16.5% and viability 94.7% with a *P*<0.05. Parks positive to *Toxocara* spp., had sports fields and playgrounds (94.4%), trees and green areas (88.8%).

**Conclusion:**

Although a low prevalence of *Toxoxara* spp. eggs in the soil of public parks was found, they exhibited high viability, suggesting that the soil from these public parks is a source of infection for pets and humans especially children.

## Introduction

*Toxocara* spp. is a cosmopolitan parasite; it infects canines and felines, causing damage in organs (lungs, brain, heart, and eyes), anemia, respiratory problems, as well as other health problems. When an infected animal coexists with humans it predisposes the occurrence of zoonotic diseases, such as human toxocariasis, mainly in children exhibiting pica (1, 2). Toxocariasis is a health problem in canines, and the feces of infected animals contain *Toxocara* spp. eggs*,* which have a great ability to survive in soil (3).

In some countries, public parks are considered as places where dogs commonly defecate (4). Several studies report *Toxocara* spp. in the soil of parks from the Iranian cities of Khorram Abad 22.2%, Ardabil 7%, Abadan 29.2%, Mashhad 9.2%, Khaf 11.3%, Karaj 36.4% and Tehran 10% (5–10), furthermore in Mexico City, there are reports of 12.9%, 18% to 39%, 63.58% according to different studies, and in Toluca 24.7% (11–14). In Sinaloa in beach sands a prevalence of 9.8% was observed (15). The presence of this nematode′s eggs represents a risk factor for the infection of its host and its dissemination throughout the city.

The objective of this study was to determine the prevalence and viability of *T. canis* eggs in the soil of public parks from Culiacan, Sinaloa.

## Material and Methods

### Study area

This study is an analytical cross-sectional observational study (16), soil sampling was carried out in parks in the city of Culiacan, Sinaloa, Mexico, located at 24°47′31 ″N and 107°23′53″ W and 60 MASL, average annual temperature of 25.7 °C, with maximum and minimum temperature of 33.2 °C and 18.3 °C respectively, and an annual rainfall of 666.1 mm (17), with a population of 905 265 inhabitants (18).

### Recovery of samples

Of 607 public parks located in the four sectors of Culiacan, a minimum sample size was determined; to achieve this task an estimation of proportions formulas was utilized (19). In each park an observational guide was performed to record the presence of sports facilities, playgrounds, sidewalks, street lighting, kiosks, benches, drinking water fountains, trees and green areas. Temperatures from Jun to Dec of 2013, were taken from the National Meteorological Agency (20). The sample size was readjusted concerning the size of the population (Table 1).

**Table 1:** Number of parks sampled and area by sector in the city of Culiacan, Sinaloa, Mexico, Country

***Sector***	***Parks (N)***	***Sampled Parks***	***Area (m^2^)***
1	85	33	173,422.77
2	66	26	130,148.58
3	286	111	413,052.39
4	170	66	205,716.71
Total	607	236	522,340.45

The Department of parks and gardens of the city of Culiacan, Sinaloa, separates the parks in 4 sectors, this division does not consider the socio-economic level of the colony, and neither contemplates the same surface in m^2^ of the parks as it indicates Table 1 (21).

Overall, 236 parks were sampled at random; in each park five soil samples were taken utilizing the sampling in a method (22). Samples were taken from an area of 10 × 10 cm and at a depth 3 cm and placed in a plastic bag (23).

The soil samples were examined at the Parasitology lab located in the Facultad de Medicina Veterinaria y Zootecnia in the Universidad Autonóma de Sinaloa.

### Detection of eggs

To detection of eggs, the centrifugation-flotation technique was used. Around 3 gr of soil sample were placed in Falcon tubes and suspended in about 6 ml distilled water. After centrifugation of the tubes test at 2500 gt for 5 min (twice), the supernatant was removed, and 10 ml distilled water was added to the tubes. After centrifugation of the tubes test as described above; the supernatant was discarded and 12 ml of zinc sulfate solution was added (ICR international group); after latter homogenized again and centrifuged, an aliquot of 15 μl of the supernatant was taken and placed on a slide. Samples of each tube were observed by optical microscope (Carl Zeiss) at the magnifications of ×10 and ×40 for *Toxocara* eggs (24).

### Viability test

The viability of *Toxocara* eggs was determined by the trypan blue staining technique (chemical reagents Hycel), from the concentrated samples obtained by flotation a sample is placed on a slide and trypan blue solution at 0.1% was added after which it is observed in an optical microscope (Carl Zeiss) at the magnification of ×10 and ×40. Stained eggs were identified as non-viable and unstained eggs as viable (25).

Identification of *Toxocara* eggs and viability of eggs found in soil of parks in each sector were analyzed with the Chi-square test (26).

### Statistical analysis

The installations present at every park are presented as table of frequency, and temperature as mean value. A value *P*≤0.05 was considered significant.

## Results

**Fig. 1:**
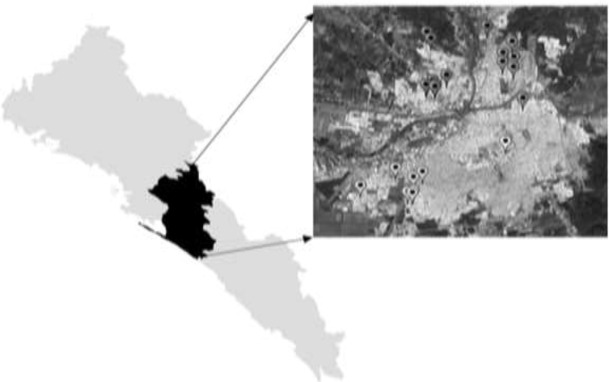
Distribution of the 18 public parks found positive to *Toxocara* spp. in Culiacan, Sinaloa, Mexico

Of 1180 soil samples obtained from 236 public parks, 18 parks tested positives to *Toxocara* spp. (*P*<0.05), with a 7.6% prevalence of parasites and viability of 94.4% (Table 2), there is a higher prevalence of *Toxocara* spp. (12.1%) in sector 1, with a distribution of positive parks in the 4 sectors of the city (Fig. 1).

**Table 2:** Prevalence and Viability of *Toxocara* eggs from positive parks by sector

***Sector***	***Sampled Parks***	***Positive parks n (%)***	***Prevalence of Toxocara in parks (%)***	***Viability of Toxocara in parks (%)***	***CI 95%***
1	33	4	12.1	100	7.9–16.2
2	26	1	3.8	100	1.3–6.3
3	111	6	5.4	83.3	2.5–8.2
4	66	7	10.6	100	6.6–14.5
Total	236	18	7.6 *P*< 0.05	94.4	4.2–11

The presence of *Toxocara* spp. in soil samples of parks is described in Table 3; the greatest presence was in sector 4 with a prevalence of 17.7% and viability of 100%. In the 18 parks that were positive to *Toxocara* spp., different types of facilities or meeting areas were observed (Table 4).

**Table 3:** Prevalence and viability of *Toxocara* spp. in soil samples from positive parks in different sectors

***Sector***	***Soil samples***	***Soil samples from positives Parks***	***Positive samples to T. canis n (%)***	***Viable eggs of T. canis n (%)***	***IC 95%***
1	165	30	13.3	100	11.3–15.2
2	130	5	20	100	17.7–22.2
3	555	35	17.1	83.3	14.9–19.2
4	330	45	17.7	100	15.6–19.9
Total	1180	115	16.5 *P*< 0.05	94.7	14.4–18.6

**Table 4:** Facilities or meeting areas present in parks positive to *Toxocara* eggs

***Types***	***Parks***	***%***
Sport fields and playgrounds	17	94.4
Trees and green areas	16	88.8
Water faucets	9	50
Walkways and lighting	9	50
Kiosks and benches	8	44.4

## Discussion

The presence and the viability of *Toxocara* spp. eggs in public parks of Culiacan, Sinaloa, can be considered a risk to public and animal health. Of the 1180 soil samples from 236 parks from 4 sectors, 115 were positives distributed in 18 public parks, which represent a 7.6% prevalence with viability of 94.4%. This could be attributed to a lack of culture on dog feces collection from visitors of these parks and the resistance of the parasite to different environmental factors.

In Tunja, Colombia (27), *Toxocara* spp. were found with a 42.5% prevalence in feces and 100% prevalence in 120 soil samples taken from 28 parks that were in an area with a temperature of 13 °C. This is due to the high numbers of parasite eggs in the soil of parks, the local weather and the lack of collection of feces by the pet owners. In Temuco, Chile (28), 193 soil samples were collected from 87 parks distributed in 6 areas of the city at a temperature of 8 to 14 °C, after analysis prevalence of *Toxocara* spp. of 12.4%, was found suggesting little sanitary care of these parks by pets’ owners. In Tenerife, Spain (4), 54 parks were sampled in 3 different zones obtaining 54 soil samples, a prevalence of *Toxocara* spp. of 37% was discovered, the authors suggested that it was due to people attitude towards feces collection by pet owners in parks and public gardens. In Abadan, Iran (7) 291 soil samples were collected from 31 parks with a prevalence of *Toxocara* spp. of 29.2%, because of the presence of stray dogs and cats in the parks. In Karaj, Iran (9), a prevalence of 36.4% of *Toxocara* spp. eggs was found, because of the high defecation of dogs and cats in the green areas of the parks.

The high prevalence found in Colombia, Chile, Spain and Iran differ from what is found in this study may be due to the number of parks and collected samples, the influence of the temperature, and lack of hygiene in the parks, factors that are a risk of contamination and dissemination of parasites. In Toluca, Mexico, a prevalence of 24.7% of *Toxocara canis* eggs was found in the soil of public parks the cold climate does not favor the development of the parasite but is favorable for its conservation (13); *T. canis* was also detected in Netzahualcoyotl (30.3%) and Tulyehualco (60%), the main causes was the high presence of dog feces, the social economic level of the area and poor maintenance of these parks (11, 12). In Coro, Venezuela a 63.16% prevalence was reported mainly due to lack of sanitary maintenance of low-income areas (29). On the other hand, in Lima, Peru the highest prevalence (63.0%) was reported in well-tended parks from the high socioeconomic stratum (2). This differs when compared with this work where positive parks were from middle-class areas that had light maintenance. In Duitama, Colombia (30), a prevalence of 34.7% was reported positive to *T. canis*, the greater prevalence, obtained could be due the low number of samples and parks sampled. In Santiago, Chile (31), a study found a prevalence of 18.2% for *T. canis*, due to different factors such as lack of maintenance, high number of stray dogs and low socioeconomic level. In Qazvin, Iran, a prevalence of 3.15% for *T. canis* was reported, the low prevalence was due to the low population of stray dogs and the prohibition of physical contact with dogs (32), in Ardabil, Iran 200 soil samples were recollected from public places in 5 zones of the city finding 35 (17.5%) of these zones positives to parasites and finding that 14 (7%) were positives to *Toxocara* spp., eggs the southern parts of the city were the most contaminated the main reasons being that inhabitants of this area are of low socioeconomic status and the easy access dogs and cats had to public places in these areas, there were also differences between seasons of year being winter and spring where it was most present (6).

In this study the same prevalence as the above-mentioned study is found, however there is differences in the number of samples and socioeconomic level but there is also easy access of these animals to the parks in the area. In Mashhad and Khaf, Iran 340 soil samples per city were collected from 39 parks in Mashhad and 29 parks in Khaf results were 195 samples from18 parks (9.2%) and 145 samples from 16 parks (11.3%) positive to *Toxocara* spp. eggs respectively (8).

Various factors (climatic, soil type, lab. methods, park cleanliness, culture, etc.), can cause variation in prevalence as well as suggesting that these areas can be a source of dissemination to animals and children. In a study in Tehran, Iran 120 public parks were tested, and 600 soil samples were taken, 12 parks (10%) were positives to *Toxocara* spp. eggs due to the low culture on feces management of the population and because *Toxocara* spp. is the most common nematode in dogs and cats in that locality (10), similar to this study where this parasite is one of the most common in the locality. In Suba, Bogotá there was a low prevalence reported (5.4%) due to temperature, vegetation, humidity and shadows areas present in the sample area that favored the survival of *Toxocara* spp. eggs in soil (23) this low prevalence coincides with what is found in this study *T. canis,* eggs showed viability of 94.4% in this study*,* suggesting a risk of infection for animals and humans by this zoonotic parasite in the city of Culiacan (Table 4). The presence of different facilities or meeting areas such as sports fields, playgrounds, trees and green areas in these parks indicates that there is a risk of infection in these soils. In an analysis in Toluca, viability of 73.3% in *Toxocara* spp. eggs was obtained (14); in Mexico City a 65.5% was reported (11) and Netzahualcoyotl a 72.6% was also found (12), the high percentages of viability in these studies may be due to *T. canis* ability to survive different climates and park cleaning. In soil from playgrounds in Kirikkale, Turkey, *T. canis* was detected showing a 62.5% prevalence making its presence a risk to children due to contamination of these areas (33), this coincides with this study in that these areas are sources of infection for children. In Urmia, Iran (34), contamination by *T. canis* in different areas such as park walkways, playgrounds and area near trash containers, was found, making these places sources for human infection this also agrees with this work where playgrounds are considered a risk zone in parks. In Tunja, Colombia (27), 42% of samples collected in green areas and playgrounds resulted positive to *T. canis*, researchers attributed this to high presence of eggs in the environment, differing with this study where a low presence with high viability of *T. canis* eggs was found. In Guarulhos, Brazil (35), the public parks of 47 districts were examined and 35 were contaminated by parasites with a prevalence of *Toxocara*. spp. 68.1%, in the district that was negative, was because the good maintenance and the controlled access to the parks because the use of fences, In Lodz, Poland (36), 88 samples from 22 sites between play areas and sandpits were examined, they found *Toxocara* eggs in the 40.1% of the sandpits and 50% in the play areas, in the places that were secured from the access of animals show less eggs of *Toxocara* that the places no secure. In Erzurum, Turkey (37), 214 soil samples were collected form 36 public parks which 28 were unfenced and 8 fenced, they found a prevalence of *Toxocara* spp. of 64.28% in the unfenced parks and found nothing in the fenced parks if we compared the parks of Culiacan with the parks of Brazil, Poland and Turkey, we noticed that the majority are unfenced and the ones fenced were unlocked give the animals stray or with owner access all the time, despite that with have a low prevalence of *Toxocara* in comparison with the other works, this show that the parks of Culiacan need more protection to reduce or eliminated the presence of parasites in the parks.

The temperature needed for *T. canis* eggs to develop after being excreted in feces is from 25 to 30 °C, these temperatures may vary depending on soil type and park conditions (38). In 5 states in Peninsular Malaysia (39), were collected 300 samples of 60 playgrounds in the wet and dry season they found contaminated with *Toxocara* spp. (95.7%), the presence of eggs were more prominent in the wet season that the dry season because the optimum temperature, high levels of humidity and moisture in the soils enhance the survival and viability of the parasites, in this study, the average temperature was between 29–31 °C in comparison with the work in Malaysia that has a ranging temperature between 25.5–28.6 °C (39), this show that Culiacan environment is ideal for the development of *Toxocara* spp. eggs, but the reason that we have a low prevalence in comparison with Malaysia it due to another factor like the human and stray animals defecation in the parks that combinate with their climate make the survival of the parasites in their parks more successful.

The factors that influence the presence of *T. canis* in the soil of public parks, such as climate, canine population, maintenance, culture, region, sample area, sample collection method, sample processing method, viability and infectivity determine infection capacity and dissemination of the parasite to pets and humans.

## Conclusion

Although a low prevalence of *Toxoxara* spp. eggs in the soil of public parks were found, they exhibited high viability, suggesting that the soil from these public parks is a source of infection for pets and humans especially children.
